# Supplementary material for: ADAR1 mediated regulation of neural crest derived melanocytes and Schwann cell development
Source: Nat Commun. 2020 Jan 10;11:198. doi: 10.1038/s41467-019-14090-5 (PMC6954203; doi:10.1038/s41467-019-14090-5)
Supplement: Supplementary file 1 — Supplementary Information [file 41467_2019_14090_MOESM1_ESM.pdf]

Supplementary Information

for

ADAR1 mediated regulation of neural crest derived melanocytes and Schwann cell development

by

Nadjet Gacem et al.

SUPPLEMENTARY FIGURES

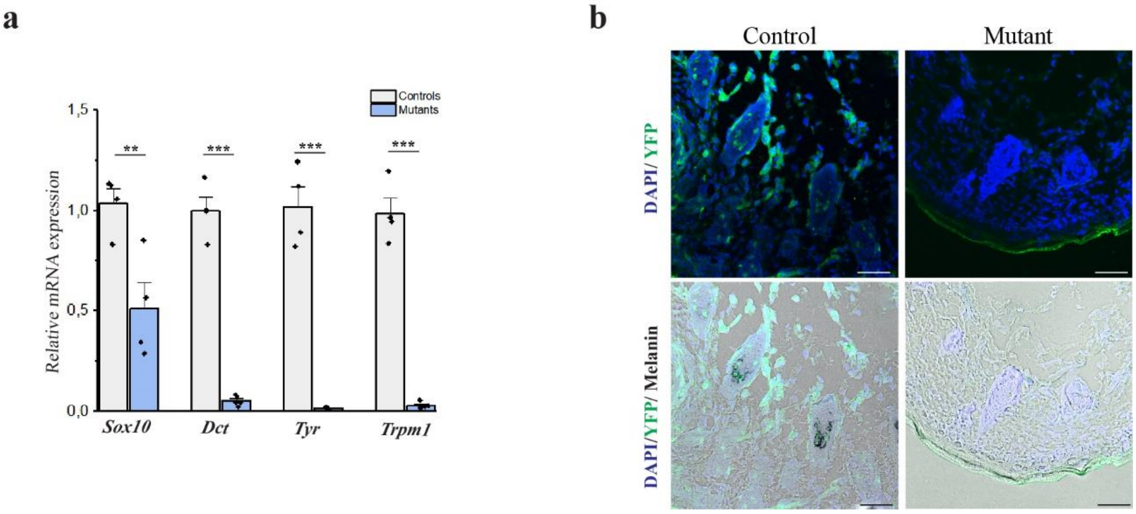

Supplementary Figure 1. Wnt1-Cre mediated *Adar1* deletion leads to reduced melanocytes numbers at birth. (a) Relative expression of melanocytes expressed genes in the skin of newborn control (grey) or mutant (blue) mice (n = 4 independent controls and n=4 independent mutants) analyzed by RT-qPCR. All samples were normalized to β-actin and expression of each amplicon in mutants and controls expressed relative to controls. Data represent mean ± SEM, asterisks represent P values: **$p < 0.01$, ***$p < 0.001$ determined using t-test. Note the severe reduction in the relative expression of melanocyte markers tested in mutants compared to controls. Source data are provided as a source data file. (**b)** Skin sections of mutants and controls immunostained with YFP. Representative regions showing YFP (green) cells in hair follicles of control and mutant mice are presented. Note the drastic reduction of YFP-positive/melanin producing cells within hair follicles of mutants compared to controls. Melanin (black) is shown on white light images. Counter staining with DAPI (blue) is shown to identify structures. Scale bar: 50μm.

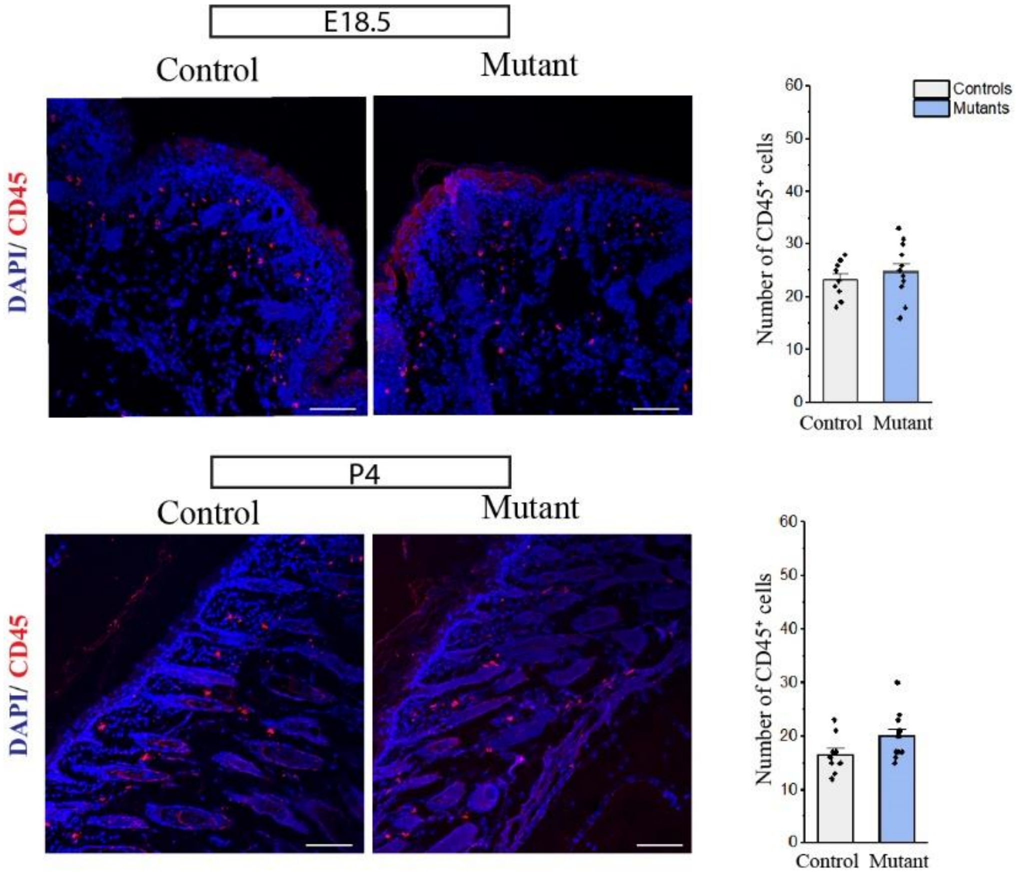

Supplementary Figure 2. Immune cells in skin of mutants and controls at E18.5 and P4. Skin sections of *HtPA-Cre; Adar1* $^{fl/fl}$ mutants and controls immunostained with CD45 (red). Representative regions along with quantification of the number of positive cells per sections are presented at both stages. Counter staining with DAPI (blue) is shown to identify structures. Scale bar: 100µm. Statistical differences between the groups (counts performed on n=9 independent sections of 3 controls in grey and n=12 independent sections of 3 mutants in blue) are represented as mean ± SEM, t-test performed found non-significant differences. Source data are provided as a source data file.

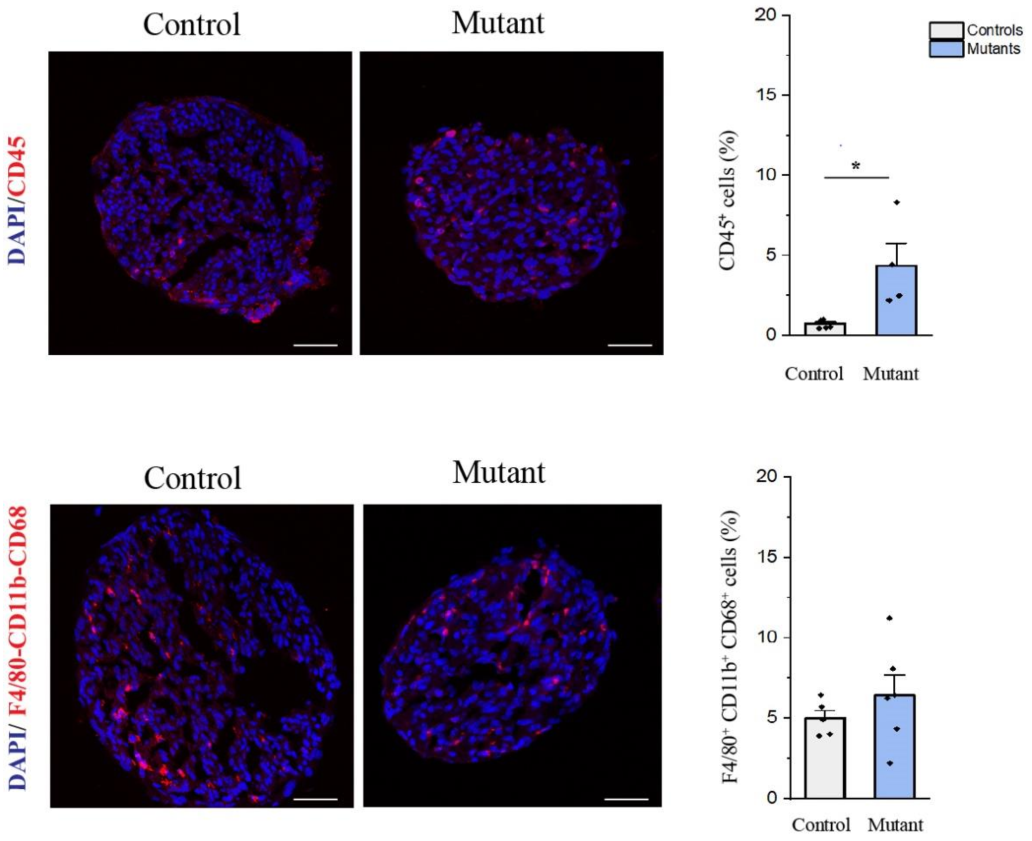

Supplementary Figure 3. Immune cells in sciatic nerves of mutants and controls at P4. Sciatic nerves sections of *HtPA-Cre; Adar1* $^{fl/fl}$ mutants and controls immunostained with CD45 (red) or a cocktail of (F4/80+CD11b+CD68, red) are presented. Counter staining with DAPI (blue) is shown to identify structures and count cells. Representative regions along with quantification (mean ± SEM) of the % of red positive cells/DAPI are presented. Statistical differences between the groups (counts performed on n=5 independent sections of 3 controls in grey, n=4 independent mutants for CD45 cells and n=6 independent sections of 4 mutants for F4/80+CD11b+CD68 cells in blue) were determined using t-test, Asterisk represent P value: $*p < 0.05$). Note non-significant differences observed when using F4/80+CD11b+CD68 antibody and the small but significant difference observed using CD45. Scale bar: 50µm. Source data are provided as a source data file.

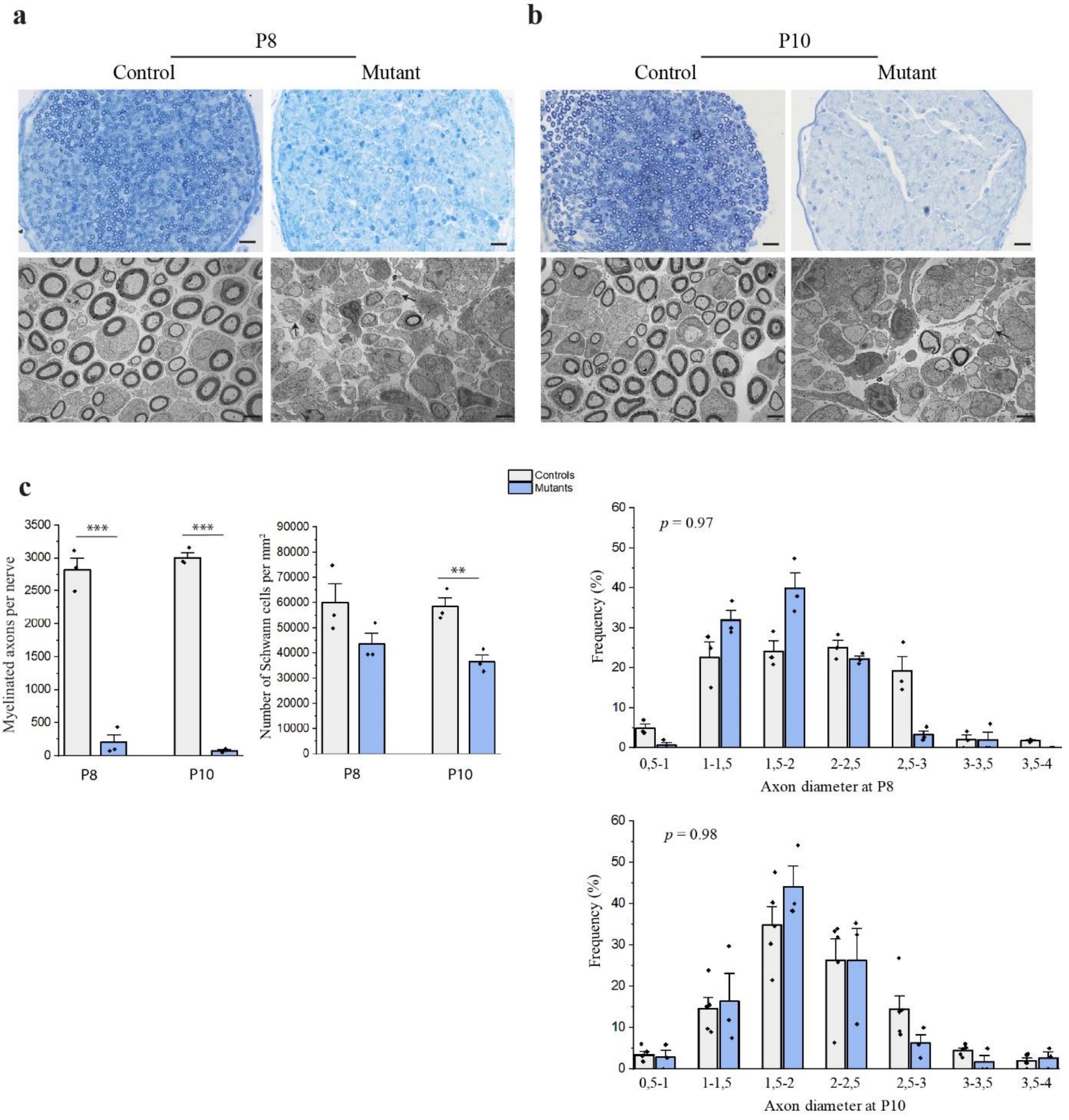

Supplementary Figure 4. Analysis of sciatic nerves of mutants at P8 and 10. (a-b) Toluidine blue-staining and electron micrographs of transverse sections of sciatic nerves from control and *HtPA-Cre; Adar1* $^{fl/fl}$ mutant mice at P8 (**a**) and P10 (**b**). Note the severe reduction of myelin sheaths in the mutants compared to controls. Scale bars: 10µm (toluidine staining) / 2µm (electron micrographs). (**c**) Quantifications performed from TEM pictures to count myelinated axons number per nerve at P8 and 10 (n=3 mice/group), SCs number per square millimeter at P8 and 10 (n=3 mice/group), distribution of axon diameter at P8 (n=3 mice/group) and distribution of axon diameter at P10 (n=5 controls and n=3 mutants). Controls are indicated in grey and mutants in blue. In all graphs presented in (**c**) graphs represent mean ± SEM. Statistical differences between the groups was determined using t-test (Asterisks represent P value: $^{**}p < 0.01$ and $^{***}p < 0.001$). For axon diameter statistical analysis, Kolmogorov–Smirnov test was performed and P value indicated on the respective panels. Source data are provided as a source data file. Note the drastic decrease in the number of myelinated axon profiles per nerve section, the significant (although modest) reduction in the number of SCs observed at P10 only, and the absence of axons diameters differences observed in mutants compared to controls.

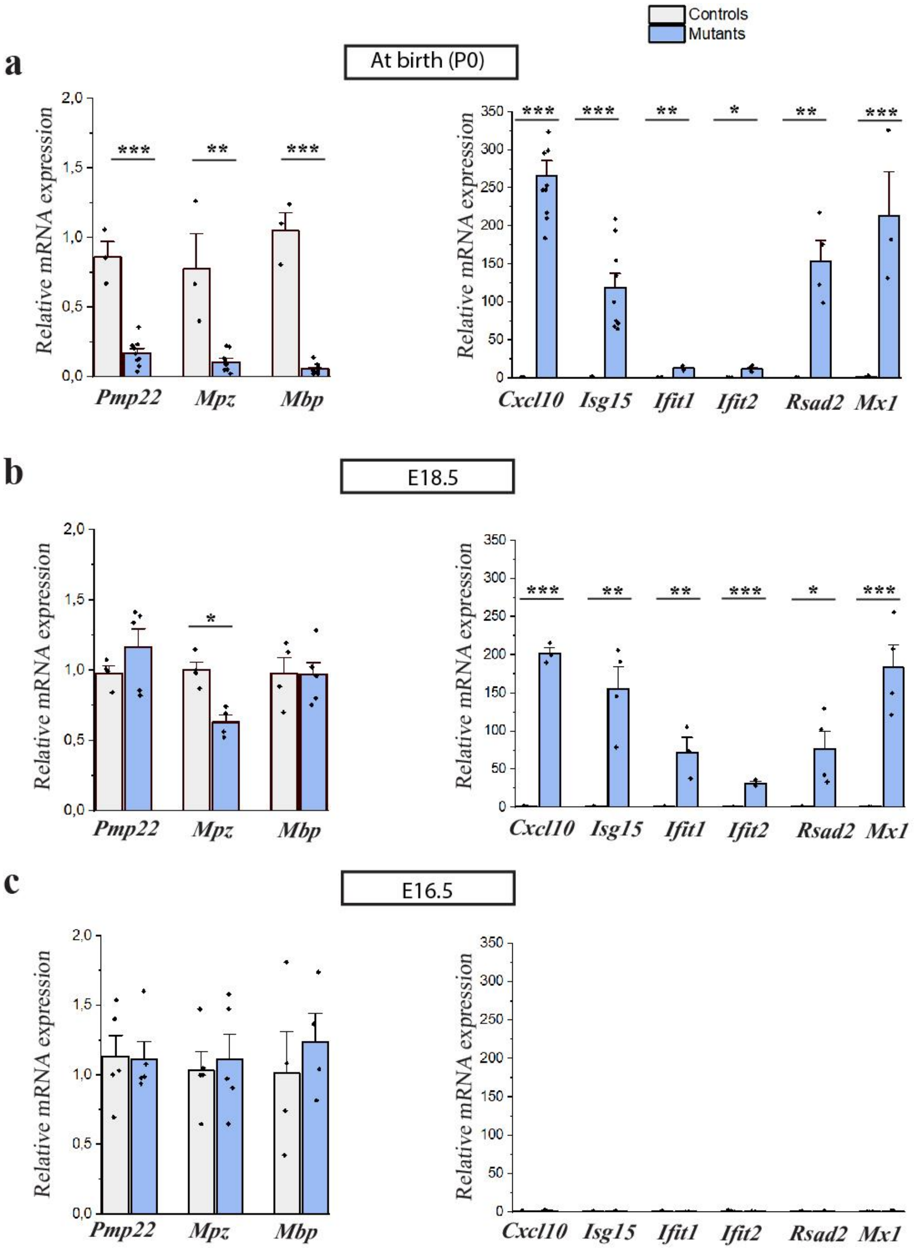

Supplementary Figure 5. ADAR1 controls SCs myelination from birth. Relative expression of Schwann cell markers (*Pmp22, Mpz, Mbp*) and Interferon stimulated genes (*Cxcl10, Isg15, Ifit1, Ifit2, Rsad2, Mx1*) in sciatic nerves harvested from controls (grey) and *HtPA-Cre; Adar1* $^{fl/fl}$ mutants (blue) at birth (P0) (**a**), E18.5 (**b**) and E16.5 (**c**) analyzed by RT-qPCR. For ISGs, n=3 controls and n=3 or 7 mutants were compared, depending on the genes. For myelin genes, n=3 to 5 independent controls and n=4 to 7 independent mutants were compared, depending on the genes. Source data are provided as a source data file. All data represent mean ± SEM. Statistical differences between the groups were determined using t-test, Asterisks represent P values: **p* <0.05, ***p* < 0.01, ****p* < 0.001.

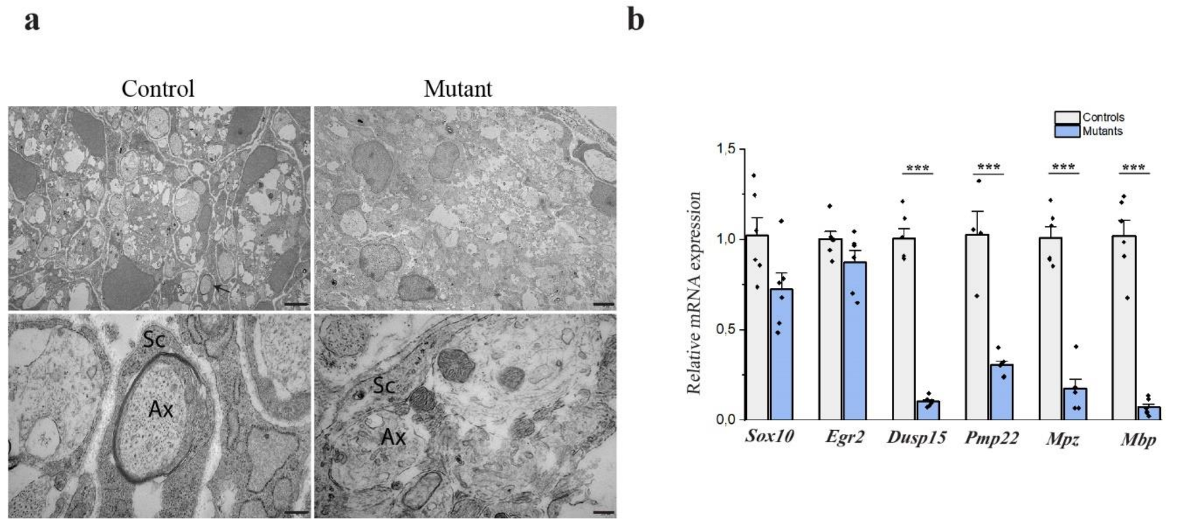

Supplementary Figure 6. *Wnt1-Cre; Adar1* $^{fl/fl}$ sciatic nerves are devoided of myelin sheaths at birth. (a) Electron micrographs of transverse sections of sciatic nerves from control and *Wnt1-Cre; Adar1* $^{fl/fl}$ mutant mice at birth. Note the lack of myelin initiation in the mutants relative to controls. Arrow indicate Schwann cells starting their myelination process in controls. Note that analysis was limited by the early lethality of these mutants, which all died at birth, i.e at the very early onset of myelin formation. Scale bars: 2µm upper panels / 500nm for insets presented in lower panels. Ax: axon; Sc: Schwann cells. (**b)** Relative expression of SCs differentiation markers analyzed by RT-qPCR on RNAs extracted from isolated sciatic nerves of control (grey) and mutant (blue) mice. Data represented as mean ± SEM. n= 6 independent controls and n=6 independent mutants for all genes except *Pmp22* for which n=4 controls and n=3 mutants were analyzed. Statistical differences between the groups were determined using t-test (asterisks represent P value: $***p < 0.001$). Source data are provided as a source data file.

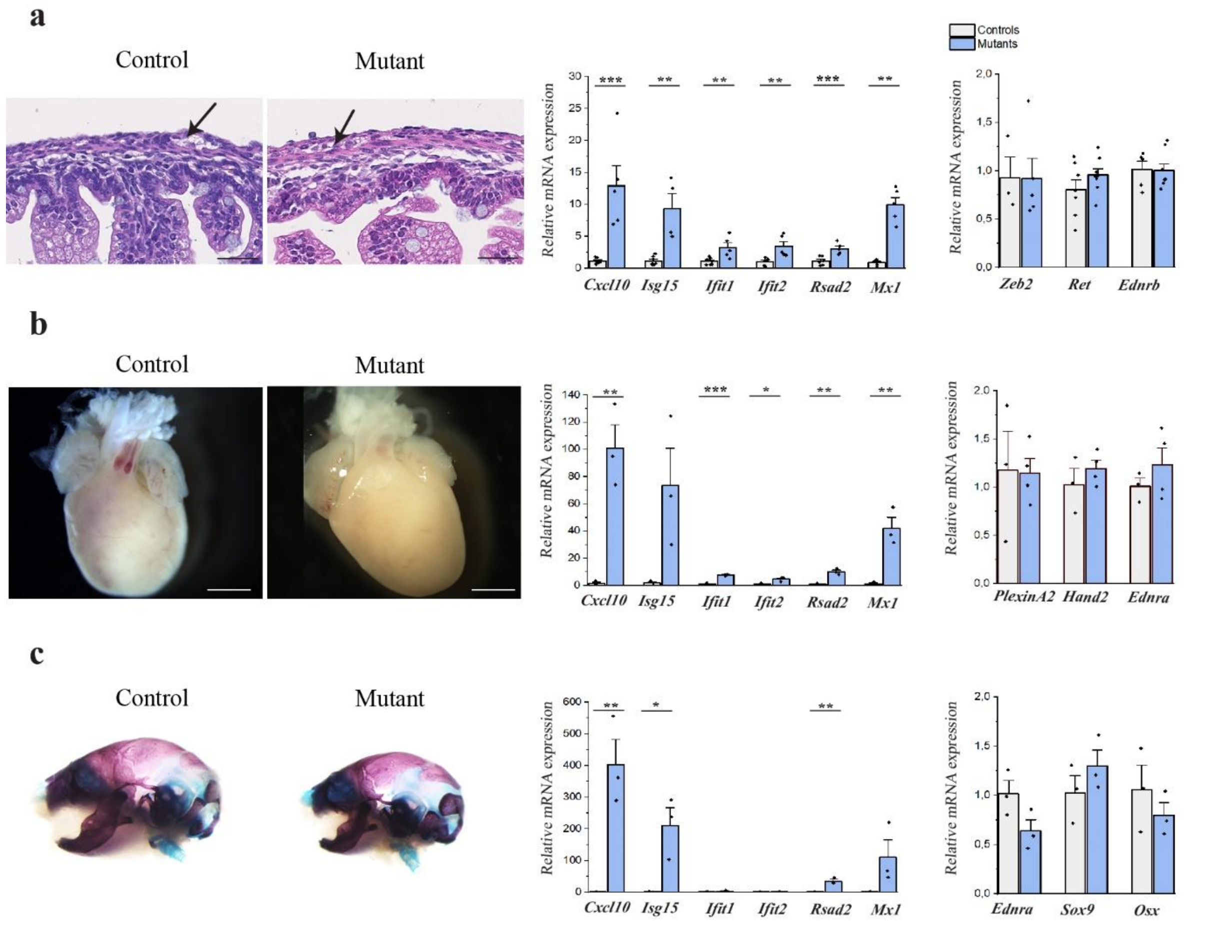

Supplementary Figure 7. Analysis of (a) enteric nervous system, (b) cardiac, (c) cranio-facial structures of mutants versus controls at P4. Left columns represent sections of gut from control and *HtPA-Cre; Adar1* $^{fl/fl}$ mutant mice stained with Hematoxylin and eosin (H/E) (Scale bar: 50µm), general view of heart (Scale bar: 1mm) and lateral view of skull of control and *Adar1* mutant upon Alizarin Red and Alcian Blue staining. Although stomach of mutants are smaller, enteric ganglia (Black arrowhead) are present in normal position along the whole length of the gut (**a**). Analysis of the heart revealed no major alterations either (**b**, no outflow tract defects). Although heads of mutants were smaller (but correlate with global size reduction of mutant pups), no specific facial alterations were noticed (**c**).

Right columns represent relative expression level of Interferon stimulated genes (ISG signature composed of *Cxcl10, Isg15, Ifit1, Ifit2, Rsad2, Mx1*) and expression of 3 NC expressed genes (*Zeb2, Ret Ednrb* for gut; *PlexinA2*, *Hand2*, *Ednra* for heart; *Ednra*, *Sox9* and *Osx* for mandible) quantified in mutant (blue) relative to control (grey) gut (**a**), heart (**b**) and mandible (**c**) tissues. The relative abundance values of each amplicon was normalized to the internal control β-actin, and expression level of mutants and controls represented relative to controls. All data represent mean ± SEM, (for (**a**): n= 4 or 5 controls and n=4 mutants depending on the tested genes; for (**b**) n= 3 controls and n=3 or 4 mutants depending on tested genes; for (**c**) n=3 controls and n=3 mutants) asterisks represent P values: $*p < 0.05$, $**p < 0.01$, $***p < 0.001$ determined using t-test. Source data are provided as a source data file.

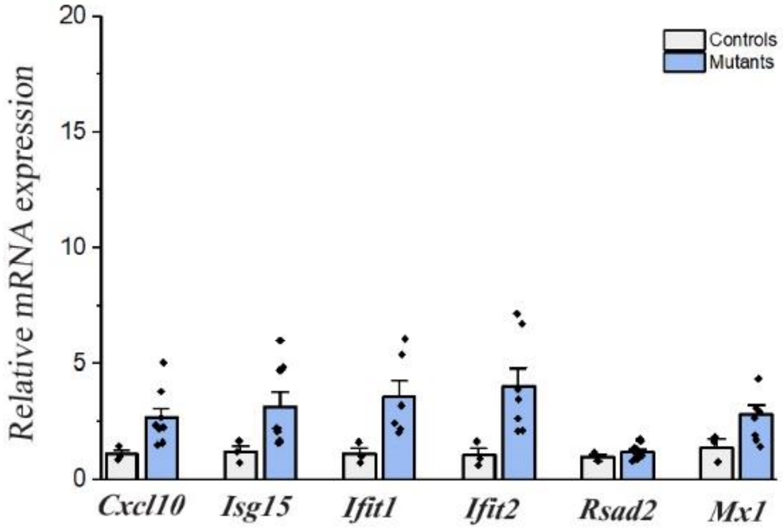

Supplementary Figure 8. Analysis of ISGs signature in liver of mutants and controls. Relative expression of Interferon stimulated genes (*Cxcl10*, *Isg15*, *Ifit1*, *Ifit2*, *Rsad2*, *Mx1*) in livers harvested from post natal day 4 controls (grey) and *HtPA-Cre; Adar1* $^{fl/fl}$ mutants (blue) analyzed by RT-qPCR. All data represent mean ± SEM. Statistical analysis between the groups (n=8 independent comparisons of 3 controls and 3 mutants) were determined using t-test and were found non-significant. Source data are provided as a source data file. Note the absence of systemic response.

SUPPLEMENTARY TABLES

Supplementary Table 1. List of genes involved in EMT process found deregulated ≥ 2fold in *Adar1* mutants relative to controls

Gene Symbol	Gene Name	Ratio	Adjusted P-Value
Shh	sonic hedgehog	102,82	4,33E-04
Timp1	tissue inhibitor of metalloproteinase 1	14,23	2,68E-67
Tnc	tenascin C	11	6,71E-108
Hbegf	heparin-binding EGF-like growth factor	7,41	1,25E-111
Plaur	plasminogen activator, urokinase receptor	7,32	1,82E-32
Myc	myelocytomatosis oncogene	7,13	1,62E-69
Igfbp3	insulin-like growth factor binding protein 3	6,86	1,07E-134
Hpse	heparanase	6	7,19E-22
Anxa1	annexin A1	5,37	3,44E-44
Cd44	CD44 antigen	4,01	1,29E-34
Axl	AXL receptor tyrosine kinase	3,73	7,30E-53
Vcan	versican	3,49	4,26E-04
Hspb1	heat shock protein 1	3,37	9,68E-11
Cxcl16	chemokine (C-X-C motif) ligand 16	2,97	1,91E-08
Cyr61	cysteine rich protein 61	2,84	3,04E-22
Braf	Braf transforming gene	2,65	5,59E-05
Hmga2	high mobility group AT-hook 2	2,6	4,22E-17
Eng	endoglin	2,31	7,00E-22
Itga5	integrin alpha 5 (fibronectin receptor alpha)	2,3	5,88E-16
Pde4a	phosphodiesterase 4A, cAMP specific	2,2	4,18E-04
Flt1	FMS-like tyrosine kinase 1	2,12	1,01E-09
Zfp217	zinc finger protein 217	2,06	1,74E-06
Met	met proto-oncogene	2,03	1,13E-06
Tgfb1i1	transforming growth factor beta 1 induced transcript 1	2,03	2,53E-10
Trps1	trichorhinophalangeal syndrome I (human)	2,02	4,74E-09
Map3k4	mitogen-activated protein kinase kinase kinase 4	0,49	6,17E-12
Dab2	disabled 2, mitogen-responsive phosphoprotein	0,47	4,58E-12
Tgfa	transforming growth factor alpha	0,45	1,20E-06
Ptn	pleiotrophin	0,44	9,50E-12
Eif5a2	eukaryotic translation initiation factor 5A2	0,38	7,48E-23
Kras	Kirsten rat sarcoma viral oncogene homolog	0,38	1,36E-22
Col8a2	collagen, type VIII, alpha 2	0,36	6,03E-03
Fgf1	fibroblast growth factor 1	0,35	2,08E-35
Itga6	integrin alpha 6	0,35	1,91E-20
Itgb4	integrin beta 4	0,32	3,68E-28
Bmp7	bone morphogenetic protein 7	0,26	7,42E-12
Ndrg1	N-myc downstream regulated gene 1	0,26	7,31E-26
Mgat3	mannoside acetylglucosaminyltransferase 3	0,22	4,61E-07
Cdh1	cadherin 1	0,20	4,34E-02
Wnt6	wingless-type MMTV integration site family, member 6	0,16	3,19E-33

Supplementary Table 2: List of primers sequences

Genotype primers		
Adar1$^{fl/fl}$	Forward	AGGTGGAGAATGGTGAGTGG
	Reverse	GCACTGGAGGACAGAAGAGG
	Lox	CAGGTCGGTCTTGACAAAAAG
R26R	YFP1	AAAGTCGCTCTGAGTTGTTAT
	YFP2	GCGAAGAGTTTGTCCTCAACC
	YFP3	GGAGCGGGAGAAATGGATATG
HtPA-Cre	Forward	TGTCTCCTCTTCTTTCTCTTA
	Reverse	CGCCTGAAGATATAGAAGATA
Wnt1-Cre	Forward	GCCTGCATTACCGGTCGATGC
	Reverse	CAGGGTGTTATAAGCAATCCCC

Supplementary Table 3: List of primers sequences forward and reverse from 5'-3' used for RT-qPCR

	RT-qPCR primers				
	**Genes**				
	b actin	Forward	CCCTCACGCCATCCTGCGTC	Reverse	GCGGCAGTGGCCATCTCCTG
	Sox10	Forward	CAACCACCCCAAAGACAGAG	Reverse	TTGGTCCAGCTCAGTCACAT
Pigmentation signature	***Mitf-M***	Forward	GCCTTGTTTATGGTGCCTTC	Reverse	GTCCTCCTCCCTCTACTTTCTGT
	Dct	Forward	CAACTGCAGCGTGTATGACT	Reverse	GTTCTTCCCGGTTGCAAAGT
	Trpm1	Forward	ACCATGTCCAACCCTCTGAG	Reverse	GAAACCACGTTAGGACCACC
	Tyr	Forward	AGCAGATGTGGAATTTTGTCTGA	Reverse	CCACAAAAGCATGGTGAAGAAG
Myelination	***Erbb3***	Forward	AGCGACACAGCCTGCTTA	Reverse	TCGGTACCCAGCACAGAACT
	Egr2	Forward	GCCCCTTTGACCAGATGAAC	Reverse	AGGGTACTGTGGGTCAATGG
	Dusp15	Forward	ACTCCTGAGGTACCCATCAA	Reverse	TCCAGTCACCGTCATCACAT
	Pmp22	Forward	GTTCCTGTTCTTCTGCCAGC	Reverse	CGAAGCCATAGGAGTAGTCAGT
	Mpz	Forward	AGACTACAGTGACAACGGCA	Reverse	AGAAGAGCAACAGCAGCAAC
	Mbp	Forward	CTCTGGCAAGGACTCACACA	Reverse	TGTCTCTTCCTCCCCAGCTA
	Ugt8a	Forward	TGACTAGAGTACAGGCAAAAGG	Reverse	TGATGGACAGCAGAACGGAG
	Fyn	Forward	GACCTCCATCCCGAACTACA	Reverse	ATCTTCCGTCCGTGCTTCA
	Plp1	Forward	GTATGGCTCCTGGTGTTTGC	Reverse	GCAGATGGACAGAAGGTTGG
	Hes1	Forward	GCACAGAAAGTCATCAAAGCC	Reverse	CGGTATTTCCCCAACACGC
Denervation	***Vgf***	Forward	GCAGGGAAAACTTCGGCTAC	Reverse	GAGCTCTACGTATCGGTGGA
	Egr1	Forward	AGCGAACAACCCTATGAGCAC	Reverse	TCGTTTGGCTGGGATAACTCG
	Fgf1	Forward	AGTGCGGGCGAAGTGTATAT	Reverse	CTTCTTGAGGCCCACAAACC
	Fgf5	Forward	GATCTACCCGGATGGCAAAG	Reverse	CAATCCCCTGAGACACAGCA
	Atf3	Forward	GAGATGTCAGTCACCAAGTC	Reverse	CAGTTCTCTGACTCTTTCTGC
	Lgals3	Forward	TGGGGAAAGGAAGAAAGACA	Reverse	TCATCCGATGGTTGTACTGC
	Btc	Forward	AATTCTCCACTGTGTGGTAGCA	Reverse	GGTTTTCACTTTCTGTCTAGGGG
	Drp2	Forward	GCATCGCCACATTGAGCATA	Reverse	ATCAGAGATGGCAAGCTGGT
	Wif1	Forward	CACTGCAATAAGAGGTATGGAGC	Reverse	GGGTTCACCAGATGTAATTGGA
	Ednrb	Forward	TCCTCGTGTTTGCTCTCTGT	Reverse	CCAGCAGCACAAACATGACT
	Igfbp2	Forward	ACCCCTTGCCAGCAGGAGTTGGA	Reverse	TCCCTGGATGGGCTTCCCGGT
	Hmga2	Forward	CAGCAGCAAGAGCCAACCTG	Reverse	TGTTGTGGCCATTTCCTAGGT
	Runx2	Forward	CGGCCCTCCCTGAACTCT	Reverse	TGCCTGCCTGGGATCTGTA
	Tnc	Forward	GAGGACTTCTATCGCAACTGG	Reverse	CTTGGGCTGTGATTTTGCTC
	Tfap2a	Forward	TGGTAAACCCCAACGAAGTC	Reverse	GTTGGCAGCTTTACGTCTCC
	Shh	Forward	ACTATGAGGGTCGAGCAGTG	Reverse	CGTAAGTCCTTCACCAGCTTG
	Gdnf	Forward	CTTGGGTTTGGGCTATGAAA	Reverse	ACAGGAACCGCTGCAATATC
ISG Signature	***Cxcl10***	Forward	CTTGATGGTCTTAGATTCCGGATTC	Reverse	AAGTGCTGCCGTCATTTTCTGCCTC
	Isg15	Forward	GGTGTCCGTGACTAACTCCAT	Reverse	TGGAAAGGGTAAGACCGTCCT
	Ifit1	Forward	GCCATTCAACTGTCTCCTG	Reverse	GCTCTGTCTGTGTCATATACC
	Ifit2	Forward	AGTACAACGAGTAAGGAGTCACT	Reverse	AGGCCAGTATGTTGCACATGG
	Rsad2	Forward	TGCTGGCTGAGAATAGCATTAGG	Reverse	GCTGAGTGCTGTTCCCATCT
	Mx1	Forward	GACCATAGGGGTCTTGACCAA	Reverse	AGACTTGCTCTTTCTGAAAAGCC
Other NC derivatives	***zeb2***	Forward	CCGCCACGAGAAGAATGAAG	Reverse	GGTGCTTGTACTTGAAGGCC
	Ret	Forward	CATGTTACCCGTGCAGTTCC	Reverse	GTCTCTTTGTTGGCACCAGG
	Ednrb	Forward	TCCTCGTGTTTGCTCTCTGT	Reverse	CCAGCAGCACAAACATGACT
	PlexinA2	Forward	CACATGACCCCACTACCTGT	Reverse	CACATGACCCCACTACCTGT
	Hand2	Forward	CAGCTACATCGCCTACCTCA	Reverse	CAGCTACATCGCCTACCTCA
	Ednra	Forward	GGGCATCACCGTCTTGAA	Reverse	GGAAGCCACTGCTCTGTACC
	Sox9	Forward	GACTCCCCACATTCCTCCTC	Reverse	CTGCTCAGTTCACCGATGTC
	Osx	Forward	CTCACTATGGCTCCAGTCCC	Reverse	AGGTGGGTAGTCATTTGCA
