# Supplementary material for: ADAR1 mediated regulation of neural crest derived melanocytes and Schwann cell development
Source: Nat Commun. 2020 Jan 10;11:198. doi: 10.1038/s41467-019-14090-5 (PMC6954203; doi:10.1038/s41467-019-14090-5)
Supplement: Supplementary file 3 — Description of Additional Supplementary Files [file 41467_2019_14090_MOESM3_ESM.pdf]

Description of Additional Supplementary Files

File name: Supplementary Data 1

Description: List of genes found deregulated ≥ 2fold in *Adar1* mutants relative to controls.

File name: Supplementary Data 2

Description: List of genes involved in repair process found deregulated ≥ 2fold in *Adar1* mutants relative to controls. The last column shows results from references 39 and 40. J1 to J7 indicates the day post injury and the last column indicates if genes are within the interferome database.

File name: Supplementary Data 3

Description: List of edited sites found in controls and reduced in mutants. Selection criteria were 1) Min total coverage in all samples > 10; 2) A>G variant only (RNA level); 3) Not in dbSNP142; and 4) p value <0.1. Each of the columns represent Chromosome position, position using reference mm10 genome, the modification, their previous identification in different databases (1 yes, 0 no), the mean cover of concerned regions and the mean % of editing, the p value, the % of editing within each samples (controls C or mutants M), the consequences of each modification, the name of the gene and the expression profile of the gene compared to RNAseq data presented (up: upregulated, down:downregulated). Presence in the Interferome database is noted IRG.
